# Sequences Related to Chimay Rhabdovirus Are Widely Distributed in *Ixodes ricinus* Ticks across England and Wales

**DOI:** 10.3390/v16040504

**Published:** 2024-03-26

**Authors:** Mirjam Schilling, Megan Golding, Ben P. Jones, Karen L. Mansfield, Sara Gandy, Jolyon Medlock, Nicholas Johnson

**Affiliations:** 1 Vector-Borne Diseases Workgroup, Animal and Plant Health Agency, Woodham Lane, Addlestone KT15 3NB, UKnick.johnson@apha.gov.uk (N.J.); 2 Rabies and Viral Zoonoses Workgroup, Animal and Plant Health Agency, Woodham Lane, Addlestone KT15 3NB, UK; 3 Medical Entomology and Zoonoses Ecology, UK Health Security Agency, Porton Down, Salisbury SP4 0JG, UK; 4 Faculty of Health and Medical Sciences, University of Surrey, Guildford GU2 7XH, UK

**Keywords:** tick, *Ixodidae*, rhabdovirus

## Abstract

Ticks are the main arthropod vector of pathogens to humans and livestock in the British Isles. Despite their role as a vector of disease, many aspects of tick biology, ecology, and microbial association are poorly understood. To address this, we investigated the composition of the microbiome of adult and nymphal *Ixodes ricinus* ticks. The ticks were collected on a dairy farm in Southwest England and RNA extracted for whole genome sequencing. Sequences were detected from a range of microorganisms, particularly tick-associated viruses, bacteria, and nematodes. A majority of the viruses were attributed to phlebo-like and nairo-like virus groups, demonstrating a high degree of homology with the sequences present in *I. ricinus* from mainland Europe. A virus sharing a high sequence identity with Chimay rhabdovirus, previously identified in ticks from Belgium, was detected. Further investigations of *I. ricinus* ticks collected from additional sites in England and Wales also identified Chimay rhabdovirus viral RNA with varying prevalence in all tick populations. This suggests that Chimay rhabdovirus has a wide distribution and highlights the need for an extended exploration of the tick microbiome in the United Kingdom (UK).

## 1. Introduction

Ticks are highly specialised obligate hematophagous ectoparasites. There are more than 900 species of ticks described worldwide, and many of them are known to serve as pathogen vectors [1]. In Europe, *Ixodes* tick species are of high importance, as they are widely distributed, feed on a diverse range of vertebrate hosts [2] and can transmit a variety of pathogens to humans and livestock [3]. The common sheep tick, *Ixodes ricinus*, is a vector of louping ill virus and tick-borne encephalitis virus, a virus that is currently increasing in range across Europe, including in the United Kingdom (UK) [4,5]. Therefore, *Ixodes ricinus* is the subject of considerable research although many features of its biology and ecology remain unanswered. Using a metagenomics approach, studies from France, Norway, and Belgium have recently detected novel viruses within the tick virome, several of which are associated with human disease, including nairoviruses or the neurotropic Eyach virus (Reoviridae family) [6,7,8]. Although it is unclear what risk these novel viruses represent to plants, animals, or humans, the fact that they cluster phylogenetically with well-established pathogens suggests that many may have some zoonotic potential.

To investigate the composition of the microbiome of adult and nymphal *Ixodes ricinus* ticks in the UK, we collected ticks in Southwest England, a region with significantly elevated tick infestation and therefore a high risk of tick-borne diseases [9], and performed whole genome sequencing (WGS). In addition to viruses belonging to phlebo-like and nairo-like virus groups, we discovered an almost complete genome of a rhabdovirus, closely related to the Chimay rhabdovirus (genus *Betaricinrhavirus*, family *Rhabdoviridae*), first detected in *Ixodes ricinus* in Belgium [8]. An RT-qPCR screen of tick surveillance specimens using Chimay rhabdovirus-specific primers revealed a surprisingly wide distribution across the UK in both adult and nymphal stages.

## 2. Materials and Methods

Tick sampling:

The sampling location was a dairy farm, 114 hectares in size, located north of Bodmin Moor in Cornwall, Southwest England. The property consists of a series of south-facing fields, three are improved pastures, whilst the fourth is an area of rough grazing adjacent to a river. The dairy herd had repeatedly been infected with a number of tick-borne diseases [10]. Ticks were sampled from four fields in April 2021 using the flagging method and stored in EtOH before processing. All ticks collected were identified morphologically as *Ixodes ricinus*. Nymph samples were pooled into groups of 10 for processing.

Further adult *Ixodes ricinus* tick samples were obtained from a national survey of National Parks and National Landscapes (previously Areas of Outstanding National Beauty), collected between 2014 and 2018 [11]. This included samples from Dartmoor (n = 31), Exmoor (n = 20), Eryri (Snowdonia) (n = 24), and Bowland and Whitewater (North England) (n = 21). In 2023, samples from Stockland, Devon, were collected from a dairy farm, and samples from Hook, Hampshire, on heathland.

RNA extraction and whole genome sequencing:

Individual adult ticks were homogenised in 500 μL tissue culture medium using the Qiagen TissueLyser II (Qiagen, Manchester, UK) and 5 mm stainless steel beads (Qiagen, Cat. No. 69989) and centrifuged (10,000 rpm/10 min). Total RNA was extracted from 200 μL of the supernatant using TRIzol^®^ (Invitrogen, Life Technologies Limited, Paisley, UK). The precipitated RNA was re-suspended in 20 μL of nuclease-free water. RNA was extracted from the ticks collected in Devon using the All Prep DNA/RNA Mini Kit (Qiagen) following manufacturer’s instructions. RNA was extracted from the ticks from Hampshire using the QIAamp MinElute Virus Spin Kit (Qiagen), followed by DNase treatment with DNase I (Thermo Scientific, Hemel Hempstead, UK) for 30 min at 37 °C, followed by the addition of 1 µL EDTA, and enzyme denaturing for 10 min at 65 °C.

Sequencing libraries were prepared using the Nextera XT kit (Illumina, Cambridge, UK) and analysed on a MiSeq sequencer (Illumina, Cambridge, UK) with 2 × 150 base paired-end reads.

Bioinformatic analysis:

Metagenomic data analysis was performed through a cloud-based, open-source bioinformatics platform provided by the Chan Zuckerberg Initiative (CZI) [https://czid.org/, last accessed on 21 November 2023] that enables the detection of microbial pathogens from raw WGS reads [12]. Sequence analysis covered both host filtering and QC, as well as assembly-based alignment. Non-host contigs with a length of >1000 bp were selected and categorised according to their NT superkingdom name. For the ticks collected from Hampshire and Devon (2023), quality checks and trimming were performed on the raw reads using FastP [13], and Bowtie2 [14] was used to align the reads to the reference Chimay rhabdovirus sequence (MF975531). SAMtools and BCFtools [15] were then used to sort the aligned reads and create consensus sequences for phylogenetic analysis.

Phylogenetic analysis:

The obtained rhabdovirus sequences (GenBank accession number: OR853831, PP316603, PP316604) were aligned with 12 tick rhabdovirus reference sequences obtained from GenBank in Mega (v11.0.13), using Measles virus (NC_001498) as outgroup. A Bayesian phylogenetic analysis was undertaken in BEAST (v1.10.4, GTR+I+G nucleotide substitution model and 10,000,000 Markov chain Monte Carlo generations).

Chimay Rhabdovirus RT-qPCR:

A primer set was designed to amplify the 5′ end of the protein coding sequence based on the published information on Chimay rhabdovirus (GenBank accession number: MF975531) and the partial sequence obtained from *Ixodes ricinus* (GenBank accession number: OR853831). Primers, ChiRhabFor (5′-CAGACAACTTTAAACGTCAATAGG-3′) and ChiRhabRev (5-GCATAAAAGAGTCTCTTCTTGCTT-3′), were designed to amplify a 242 base pair amplicon using iTaq Universal SYBR^®^ Green One-Step kit (Bio-Rad, Hercules, CA, USA) with reverse transcriptase (Bio-Rad). The conditions used were 50 °C for 15 min, followed by a denaturation step of 95 °C for 2 min, and 45 cycles of 95 °C for 15 s, then 58 °C for 60 s. On completion, this was followed by a dissociation curve to determine the thermal melting point of the amplicon. The size of the amplicon was confirmed by separation in a 1.5% agarose gel and visualisation by staining with SYBR Safe (Invitrogen, Waltham, MA, USA) and ultraviolet illumination. Amplicons of the correct size were sequenced by Sanger sequencing using the primers ChiRabFor and ChiRabRev.

Primers Rhabdo_L_for (CTGTGGATGACATCTCCTGCCAGC) and Rhabdo_L_rev (GTGACAGCCCATGATTGAGACCTCG) were designed to amplify and characterise a 310 base pair amplicon covering the STOP codon at the 3′ end of the L segment. Successfully amplified fragments were sequenced by Sanger sequencing using the Rhabdo_L_for and Rhabdo_L_rev primers.

## 3. Results

### 3.1. Microbiomes of Ixodes ricinus Collected in a Single Location Show High Diversity

**Figure 1:**
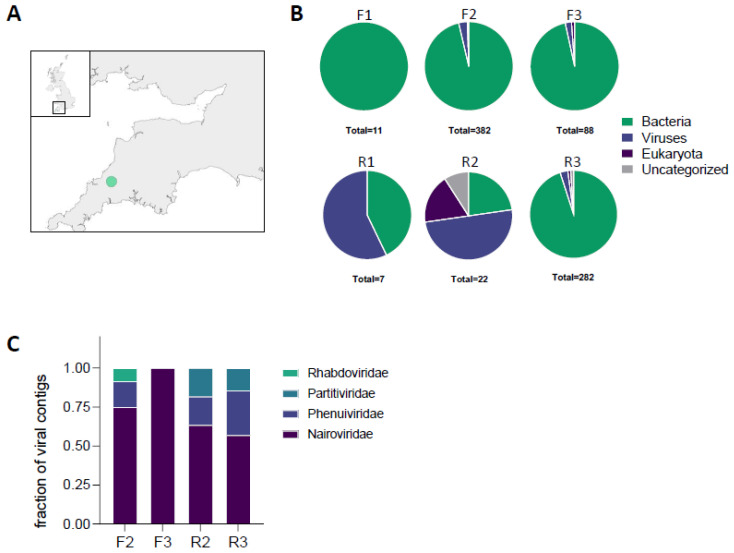
**Microbiomes of *Ixodes ricinus* collected in a single location show high diversity**. (**A**) Tick sampling site. A total of 150 *Ixodes ricinus* ticks (8 males, 7 females, and 135 nymphs) were sampled from four south-facing fields of a dairy farm located north of Dartmoor in Cornwall, UK, using the flagging method. The map was created with the sf and ggblot2 packages in R (version 4.2.3). (**B**) Illumina reads of RNA extracted from three ticks from the improved fields (F1, F2, F3) and the river field (R1, R2, R3). Shown are the number of non-host contig sequences longer than 1000 nt identified for each superkingdom in each screen. (**C**) Fraction of viral contigs longer than 1000 nt identified in each screen, categorised by their viral families.

A total of 150 ticks were sampled from the fields of a dairy farm in Cornwall (Figure 1A, Supplementary Table S1). The samples consisted of 8 males, 7 females, and 135 nymphs, all identified as *Ixodes ricinus*. Three adult female ticks were selected from fields with improved pasture (designated F1, F2, F3) and three from a field with rough grazing adjoining a river (designated R1, R2, R3). Total RNA was extracted from these six specimens and used for subsequent metagenomic analysis. Sequences for each tick sample could be attributed to a range of taxons of viral, nematode, fungal or bacterial origin (Figure 1B). The specimens exhibited a marked difference in their microbial assemblage, with one (tick F1) dominated by bacterial reads, whereas others showed the presence of a variety of microorganisms.

No viral contigs were identified for two ticks, F1 and R1. For ticks F2, F3, R2 and R3, a number of contigs matched viral sequences, the majority of which belonging to two genera, *Phlebovirus* and *Orthonairovirus*, within the order *Bunyavirales* (Figure 1C).

### 3.2. Sequences Related to Chimay Rhabdovirus Are Widely Distributed in Ticks across England and Wales

**Figure 2:**
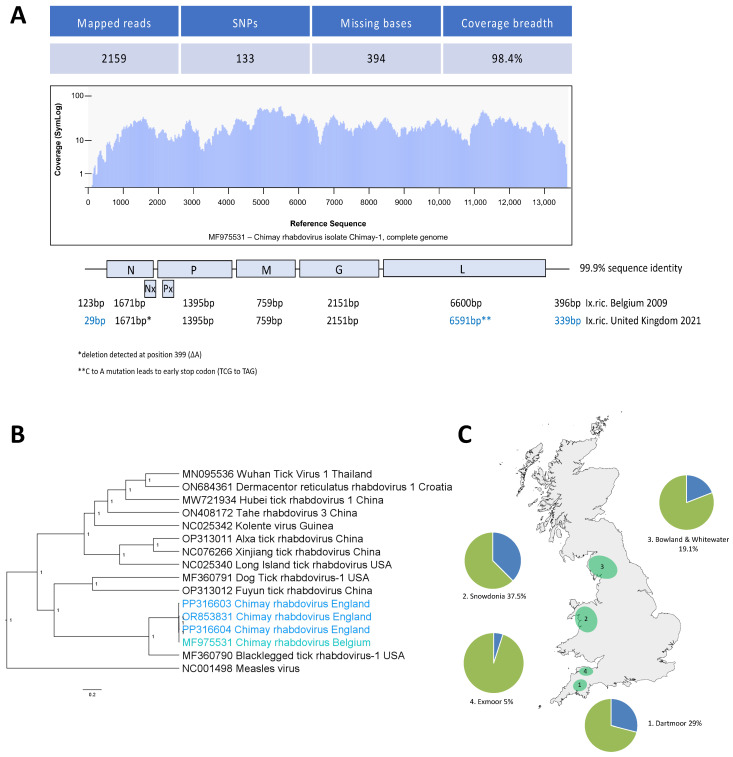
**Sequences related to Chimay Rhabdovirus are widely distributed in ticks across the UK**. (**A**) Schematic representation of the coverage of the detected rhabdovirus in tick (F2), with a genome coverage of 98.4% to the reference genome of Chimay rhabdovirus (MF975531). Using virus-specific primers, a 193 base pair fragment of the 5’ terminal of the nucleoprotein gene was obtained, producing a near complete genome. (**B**) Phylogenetic tree of the rhabdovirus sequences (GenBank accession number: OR853831, PP316603, PP316604) aligned with 12 tick rhabdovirus reference sequences obtained from GenBank in Mega (v11.0.13) using Measles virus as an outgroup. A Bayesian phylogenetic analysis was undertaken in BEAST (v1.10.4, GTR+I+G nucleotide substitution model and 10,000,000 Markov chain Monte Carlo generations). (**C**) The virus was detected by RT-PCR in 8 of 14 nymph pools collected from the same location. Further adult tick samples were collected in Dartmoor, Exmoor, Snowdonia, and the Lake District between 2014 and 2018 [11]. A screening indicated that the prevalence (blue) of Chimay rhabdovirus varied from 10% (Exmoor) to 37.5% (Snowdonia). The map was created with the sf and ggblot2 packages in R (version 4.2.3).

Additionally, in tick F2, a rhabdovirus was detected that showed a high sequence identity with a virus originally detected in *I. ricinus* ticks in Chimay, Belgium, in 2009 (GenBank accession number: MF975531), and subsequently shown to be present in ticks sampled from across Belgium [8]. The sequence’s genome coverage compared to the reference genome of the Chimay rhabdovirus was 98.4%, but it lacked a partial sequence from the 5′ terminal of the nucleoprotein gene. This was obtained by designing virus-specific primers (ChiRhabFor/ChiRhabRev) that amplified a 193 base pair fragment from the initial RNA fragment (sample F2), producing a near complete genome that included the five coding sequences present in the reference genome. The virus sequence derived in this study (OR853831) shared a high identity with that from Belgium across all gene-coding regions of the virus genome, although a point mutation within the UK virus had introduced an early stop codon in the RdRp polymerase gene (Figure 2A). When aligned with sequences of 12 other tick rhabdoviruses in a phylogeny, the identified rhabdovirus clusters with the Belgian Chimay rhabdovirus accordingly (Figure 2B, Supplementary Table S2).

In addition to the adult ticks sampled, the aforementioned primer pair (ChiRhabFor/ChiRhabRev) was used to screen pools of *I. ricinus* nymphs collected at the site in Cornwall, and the Chimay virus genome was detected in 8/14 (57.1%) of the nucleic acid samples. To investigate the distribution of Chimay rhabdovirus over a wider geographical area, nucleic acid extracts obtained from adult ticks from four other locations in England and Wales between 2014 and 2018 were tested for the presence of its genome by PCR. Chimay rhabdovirus was detected in ticks ranging back to 2014, with the prevalence of Chimay rhabdovirus varying from 5% in Exmoor to over 37% in Snowdonia (Figure 2C and Supplementary Table S3). Additionally, two near full-length Chimay rhabdovirus genomes were detected in the next-generation sequencing data from ticks collected in Hampshire (PP316604) and Devon (PP316603) in May 2023 (Figure 2B). As reported for both the Chimay rhabdovirus and blacklegged tick rhabdovirus-1, classified as betaricinrhaviruses, the Chimay rhabdovirus genomes that we detected also coded for alternative overlapping open reading frames (Nx and Px). To explore whether the early stop codon observed in the Cornwall Chimay rhabdovirus RdRp polymerase gene was also more geographically widespread, we amplified and sequenced a 310 base pair region surrounding the 3′ end of the polymerase open reading frame of 17 samples from different locations in England and Wales. Out of seven samples that amplified, one sample from Dartmoor also had an early stop codon, while another sample from Dartmoor had a synonymous mutation (TCG to TCA) at the same position. This suggests that there is a genetic flexibility tolerated around the C-terminal amino acids of the polymerase protein, with variations prevalent across the country.

## 4. Discussion

This study assessed the diversity of the microbiome of ticks (*I. ricinus*) collected from a farm in Cornwall, UK. Whole genome sequencing of the ticks revealed a range of bacterial, viral, fungal, and nematode sequences. Surprisingly, we observed a high microbiome diversity within ticks from a single location. Future studies will have to elucidate whether this is specific to the site investigated in this study, or whether this can be observed more broadly across the UK. Maybe the microbiome diversity within tick populations is linked to the diversity of host species on site? A majority of the virus sequences found in four of the ticks collected in Cornwall were identified as being related to phleboviruses and nairoviruses that have previously been detected in mainland Europe [8,16,17,18]. This study is the first report of these viruses in UK ticks.

Chimay rhabdovirus was detected in a single adult tick sample, and subsequently detected in 8 of 14 pooled samples prepared from nymphs collected from the site in Cornwall. This virus was originally detected in *I. ricinus* ticks in Chimay, Belgium, in 2009, and subsequently shown to be present in ticks sampled from across Belgium [8]. Recent studies identified the virus to be geographically as widely distributed as Bulgaria [19]. The virus sequence derived in this study shared high identity with that from Belgium across all gene-coding regions of the virus genome, including alternative open reading frames in the N and P gene. In addition to the adult ticks and nymphs sampled at the site in Cornwall, sequences of the Chimay virus genome were also detected in nucleic acid samples extracted from adult *Ixodes ricinus* from four other locations in England and Wales between 2014 and 2018, as well as in Devon and Hampshire in 2023. These findings indicate not only a wide distribution of Chimay rhabdovirus in the UK, but also suggest that there is a large group of so far uncharacterised betaricinrhaviruses yet to be discovered. Metagenomic studies on the ticks in China have generated sequences from seemingly related betaricinrhaviruses, including Mudanjiang rhabd tick virus 1 (host *Ixodes persulcatus*), Yanbian rhabd tick virus 2 (host *Haemaphysalis concinna*), and Yanbian rhabd tick virus 3 (host *Haemaphysalis japonica*), each from Jilin Province, as well as Tongren rhabd tick virus 1 (host *Haemaphysalis hystricis*) from Guizhou Province. So far, betaricinrhaviruses have not been isolated from vertebrates, and it remains elusive whether these viruses are tick specific. However, betaricinrhavirus G proteins are class I transmembrane glycoproteins that share conservation of 10–12 of the 12 conserved cysteine residues with the atypical membrane fusion G protein of vesicular stomatitis Indiana virus (species Vesiculovirus indiana) [20,21]. This conservation is typical of animal rhabdoviruses, and potentially indicates that betaricinrhaviruses might be able to infect vertebrates. Alternatively, betaricinrhaviruses might be obligately vertically transmitted, as has been shown for other insect *Rhabdoviridae* (sigmaviruses) [22]. Future studies using the Chimay rhabdovirus glycoprotein should establish whether vertebrates are seropositive for this virus, indicating a recent infection, and determine the zoonotic potential of betaricinrhaviruses.

## 5. Conclusions

This preliminary study has shown the diversity of the microbiome within ticks from a single location within the UK. Included in this are tick-associated viruses shared with *I. ricinus* from across Europe. Future work should utilise PCR analysis of *I. ricinus* ticks across the UK to confirm that the microbiome diversity reported here is not site specific or localised to Cornwall, and that the viruses detected are established within the UK. Further analysis of one of the viruses (Chimay rhabdovirus) demonstrates that it is present in both adult and nymphal stages of the species and also present in multiple locations in England and Wales.

## Data Availability

The genomic sequence for Chimay Rhabdovirus UK has been submitted to GenBank (GenBank accession number: OR853831, PP316603, PP316604).

## Supplementary Materials

The following supporting information can be downloaded at: https://www.mdpi.com/article/10.3390/v16040504/s1. Further information is provided in the Supplementary Tables S1–S3. Table S1: Summary of ticks collected during a field survey in Cornwall, UK, 2021; Table S2: Information on the host and taxonomic assignments (if available) of the rhabdoviruses used to infer the phylogenetic tree in Figure 2B; Table S3: Results of the ChiRhabFor/ChiRhabRev PCR tests for the presence of Chimay rhabdovirus RNA in ticks collected in four additional sites across England and Wales between 2014 and 2018.
