# Supplementary material for: Sequences Related to Chimay Rhabdovirus Are Widely Distributed in Ixodes ricinus Ticks across England and Wales
Source: Viruses. 2024 Mar 26;16(4):504. doi: 10.3390/v16040504 (PMC11054956; doi:10.3390/v16040504)
Supplement: Supplementary file 1 [file viruses-16-00504-s001.zip › viruses-2897717-supplementary.pdf]

Table S1. Summary of ticks collected during a field survey in Cornwall, UK, 2021.

Field	Grassland	Male	Female	Nymph
1 & 2	Improved pasture	3	0	81
3	Improved pasture	2	3	9
4 (River Field)	Rough grazing	3	4	45

Table S2. Information on host and taxonomic assignments (if available) of the rhabdoviruses used to infer the phylogenetic tree in Figure 2B.

ID	Virus	Host	Subfamily	Genus	Species
NC_001498	Measles virus		[Paramyxovirus family]	Morbillivirus	Morbillivirus hominis
MF360790	Blacklegged tick rhabdovirus-1	Ixodes scapularis	unassigned	Betaricinrhavirus	Betaricinrhavirus scapularis
MF360791	Dog Tick rhabdovirus-1	Ixodes scapularis	not available	not available	not available
MF975531	Chimay rhabdovirus	Ixodes ricinus	unassigned	Betaricinrhavirus	Betaricinrhavirus chimay
MH707450	Grenada mosquito rhabdovirus 1	Culex quinquefasciatus	not available	not available	not available
MN095536	Wuhan Tick Virus 1	Boophilus sp.	Alpharhabdovirinae	Alpharicinrhavirus	Alpharicinrhavirus wuhan
MT181988	IRE CTVM19-associated rhabdovirus	IRE/CTVM19	not available	not available	not available
MW721934	Hubei tick rhabdovirus 1	Haemaphysalis longicornis	Alpharhabdovirinae	Alpharicinrhavirus	Alpharicinrhavirus hubei
NC_025340	Long Island tick rhabdovirus	Amblyomma americanum	Alpharhabdovirinae	Sawgrhavirus	Sawgrhavirus longisland
NC_025342	Kolente virus	Amblyomma (Theileriella) variegatum/ Hipposideros jonesi (roundleaf bat)	Alpharhabdovirinae	Ledantevirus	Ledantevirus kolente
NC_076266	Xinjiang tick rhabdovirus	Hyalomma asiaticum	Alpharhabdovirinae	Lostrhavirus	Lostrhavirus hyalomma
ON408172	Tahe rhabdovirus 3	Ixodes persulcatus WZD NE-TH3	not available	not available	not available

ON684361	Dermacentor reticulatus rhabdovirus 1	Dermacento r reticulatus	not available	not available	not available
OP264902	Culex rhabdovirus	Culex pipiens	not available	not available	not available
OP313011	Alxa tick rhabdovirus	not available	not available	not available	not available
OP313012	Fuyun tick rhabdovirus	not available	not available	not available	not available

Table S3. Results of ChiRhabFor/ChiRhabRev PCR tests for the presence of Chimay rhabdovirus RNA in ticks collected in four additional sites across England and Wales between 2014 and 2018.

**Dartmoor (Southwest England)**			**Snowdonia (Wales)**		**Bowland and Whitewater (North England)**		**Exmoor**	
Year	Nr of ticks tested	Positive	Nr of ticks tested	Positive	Nr of ticks tested	Positive	Nr of ticks tested	Positive
2014	4	2			3	0	10	1
2015	6	4	4	1			2	0
2016	1	0	13	6	14	2	4	0
2017	3	1	5	2			4	0
2018	17	4	2	0	4	2		
**Total**	**31**	**11**	**24**	**9**	**21**	**4**	**20**	**1**
%		**29.0**		**37.5**		**19.1**		**5**
